# Decision‐making impairments under ambiguous and risky situations in patients with prefrontal tumor: A neuropsychological study

**DOI:** 10.1002/brb3.1951

**Published:** 2020-11-18

**Authors:** Yuyang Wang, Xukou Wang, Kai Wang, Bing Zhao, Xingui Chen

**Affiliations:** ^1^ Department of Neurosurgery the Second Affiliated Hospital of Anhui Medical University Hefei China; ^2^ Collaborative Innovation Centre of Neuropsychiatric Disorder and Mental Health Anhui Province China; ^3^ Department of Neurosurgery the Fourth Affiliated Hospital of Anhui Medical University Hefei China; ^4^ Department of Neurology the First Affiliated Hospital of Anhui Medical University Hefei China; ^5^ Anhui Province Key Laboratory of Cognition and Neuropsychiatric Disorders Hefei China

**Keywords:** brain tumor, game of dice task, iowa gambling task, prefrontal lobe

## Abstract

**Introduction:**

The neural mechanism underlying decision‐making, which is an important component of executive function, is complex and not fully understood. Few studies have directly investigated the two types of decision‐making functions – under ambiguity and under risk – in patients with brain tumors in different brain regions.

**Methods:**

Participants were classified into the ventral prefrontal cortex tumor group (VPFC, *n* = 27), the dorsolateral prefrontal cortex tumor group (DLPFC, *n* = 29), and matched healthy controls (HCs, *n* = 32). All participants were given a battery of neuropsychological tests, and they then performed the Iowa Gambling Task (IGT) and the Game of Dice Task (GDT) to assess their decision‐making under ambiguity and under risk, respectively.

**Results:**

The two patient groups performed significantly worse on attention, memory, information processing, and executive function. Additionally, patients in the DLPFC group performed significantly worse on the memory and information processing tests compared with the VPFC and HC groups.

**Conclusion:**

This study found that the decision‐making functions of participants in the VPFC and DLPFC tumor groups were impaired to varying degrees. Among them, there was decision‐making impairment under ambiguity and under risk in the VPFC group, and there was decision‐making impairment under risk in the DLPFC group.

## Graphical abstract

The neural mechanism underlying decision‐making is complicated and not fully understood. Patients with brain tumors may be a better research focus regarding decision‐making functions. Research showed that patients with prefrontal tumor showed deficits in other several cognitive and decision‐making tasks.

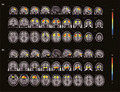

## 1 INTRODUCTION

Because of the highly developed frontal lobe, the higher cognitive functions of humans, such as learning, exploration, memory retrieval, relational reasoning, and multitasking behaviors, can be performed (Koechlin & Hyafil, 2007). As an important part of executive function, decision‐making involves many brain regions in the prefrontal cortex (Hornak et al., 2004) and has received an increasing amount of attention. Decision‐making requires several different cognitive stages (Kaiser et al., 2013), beginning with accessing relevant knowledge to create and evaluate multiple solutions or options before a decision can be made (Goel & Grafman, 2000).

According to the occurrence probability of selection results, decision‐making behaviors are divided into two categories: One is risky decision‐making, and the other category is ambiguous decision‐making (Bechara, 2004; Platt & Huettel, 2008). Previous studies have employed structured or well‐defined tasks to measure these two decision‐making behaviors to provide evidence that selective lesions to the prefrontal cortex (PFC) affect general problem‐solving functions (Baker et al., 1996). The main measurement paradigm for simulating decision‐making behavior under ambiguity is the Iowa Gambling Task (IGT), which has been used extensively in clinical research studies and shown to be a highly sensitive measure of impaired decision‐making in a variety of neurological and psychiatric conditions (Bechara, 2004; Brand et al., 2007). With this test or its modified versions, various frontal lobe‐damaged populations have been shown to exhibit decision‐making deficits, characterized by a high tendency for risky decisions on the IGT, including those with attention‐deficit/hyperactivity disorder (ADHD) (Drechsler et al., 2008), Korsakoff's syndrome (Brand et al., 2005), Parkinson's disease (PD) (Brand et al., 2004), and pathological gamblers (Labudda et al., 2007). The Game of Dice Task (GDT) permits the simulation of decision behavior under risk, with explicit and stable rules for gains and loss, and participants are able to optimize their performance by deliberately reflecting on cost–benefit analyses and calculating the expected utility. Serious impairments in the performance of the GDT have been observed in individuals with many neurological diseases, such as psychopaths, compulsive gamblers, schizophrenics, and substance abusers (Alvarenga et al., 2012). Therefore, the IGT and GDT have received increasing attention as tools to investigate the decision‐making strategies used by individuals with various neuropsychiatric disorders.

From a neuropsychological perspective, the examination of decision‐making functions is relevant in neurological/psychiatric patients because different types of anatomical or functional brain damage can lead to severe decision‐making impairments. Previous studies have shown that dysfunctions in the GDT are closely related to the dorsolateral prefrontal cortex (DLPFC), dorsal cingulate gyrus, and parietal lobe (MacDonald et al., 2000), and dysfunctions in the IGT are mediated by the limbic structures (ventral prefrontal cortex, corpus striatum, amygdala, and basal ganglia) (Bechara et al., 2003; Clark et al., 2003; Ernst & Paulus, 2005; Krain et al., 2006). However, there is increasing evidence that IGT performance is also impaired in patients with DLPFC lesions (Fellows & Farah, 2005a; Manes et al., 2002). Therefore, the underlying mechanism is complicated and has rarely been directly demonstrated.

Patients with closed‐head traumatic brain injury, severe strokes, or intracerebral hemorrhage were selected as participants with a previous impairment of prefrontal lobe decision‐making function. These participants often have extensive cognitive impairment (Gong et al., 2019; Sawamura et al., 2018), and impairments in basic cognitive functions, such as attention, memory, and learning, can seriously affect the performance of high‐level cognitive tasks (Bechara et al., 1994; Bechara et al., 1997; Hornak et al., 1996; Rolls et al., 1994). Although the area of frontal lobe damage was limited in some of the literature, the “floor effect” could not be excluded. Since the progression of brain tumors is often slow and overlooked, especially in prefrontal lobe tumors, patients frequently show no significant impairments in formal neuropsychological tests of perception, language, and intelligence but might appear markedly impaired in decision‐making; this may be a better research focus regarding decision‐making functions. Moreover, few studies have investigated the impairment of the decision‐making function in patients with tumors in different brain regions of the prefrontal cortex.

Based on the above finding, we recruited participants with prefrontal lobe tumors with relatively focal cortex damage, and we ruled out the diffuse cognitive impairments of traumatic brain injury and cerebral hemorrhage. We divided the patients into VPFC and DLPFC tumor groups to investigate decision‐making performance measured by the IGT and GDT. The aims of our study for patients with brain tumor were (a) to provide a basis to explore how damage to different brain regions in the frontal lobe affects decision‐making functions, (b) to verify the possible anatomical basis of decision‐making, and (c) to explore whether the relationship between these two types of decision‐making behaviors mediated by different anatomical structures is relatively independent or connected.

## 2 MATERIALS AND METHODS

### 2.1 Participants

Eighty‐eight participants were recruited from the First Affiliated Hospital of Anhui Medical University, and the Second Affiliated Hospital of Anhui Medical University, Hefei, China. They were assigned to three groups in the current study. The VPFC group, which included the ventromedial prefrontal cortex and orbital frontal cortex, included 27 brain tumor patients, and the DLPFC group included 29 brain tumor patients. In addition, 32 matched healthy controls participated in this study. Detailed information gathered from each participant is described in Table 1. All brain tumor patients were diagnosed by preoperative CT/MR, and the location of the tumor was in the frontal lobe, regardless of postoperative pathology. Furthermore, all participants could understand and speak Chinese. They were between the ages of 20 and 65 years and had completed higher education. None of the patient had a history of postoperative chemoradiotherapy, craniocerebral injury, visual and hearing impairment, alcohol and substance abuse, or currently suffered from psychological disorders, nor did they report the present use of psychotropic medication. All participants with subtle or severe affective disorder (HAMD > 7 and/or HAMA > 7) (Lezak, 1984) were excluded from the study to decrease interference with the neuropsychological assessment. All participants provided written informed consent and did not receive any financial or material compensation. The present study was performed in accordance with the Declaration of Helsinki and was approved by the local ethics committee.

**Table 1:** Demographic characteristics and summary of neuropsychological test of patients and healthy controls

	HC group (*n* = 32)	VPFC group (*n* = 27)	DLPFC group (*n* = 29)		ANOVA	
	Mean or count (*SD*)	Mean or count (*SD*)	Mean or count (*SD*)		*F*(2, 85)	*p*
Age (years)	51.34 (6.97)	49.59 (6.26)	48.24 (6.87)		1.633	.201
Education (years)	10.34 (1.84)	10.19 (1.67)	10.45 (2.03)		0.142	.868
HAMD	4.66 (1.21)	5.44 (1.19)	5.24 (1.43)		3.077	.051
HAMA	4.91 (1.28)	5.26 (1.53)	5.21 (1.21)		0.613	.544
MoCA	26.63 (1.85)	25.07 (2.17)^b^	24.38 (1.82)^b^		10.764	<.001
Attention/concentration						
WAIS Digit Span (forward)	5.97 (1.15)	5.85 (1.13)	5.69 (1.17)		0.449	.640
WAIS Digit Span (backward)	5.50 (0.98)	5.04 (1.06)	4.52 (1.15)^c^		6.491	.002
Stroop Color Test (sec)	16.74 (4.20)	26.21 (7.32)^b^	33.86 (5.34)^b^		69.718	<.001
Trail Making A (sec)	60.04 (11.24)	64.09 (12.26)	66.81 (10.46)		2.776	.068
Memory (AVLT)						
Immediate Recall	10.53 (1.85)	9.85 (1.70)	7.90 (2.29)^b^		14.477	<.001
Delayed Recall	9.72 (1.73)	8.74 (2.16)	6.69 (2.00)^b^		18.727	<.001
Recognition	8.81 (1.75)	7.67 (2.18)^a^	5.62 (1.80)^b^		21.623	<.001
Information Processing and Executive function						
Trail Making B (sec)	101.43 (12.52)	111.89 (11.84)^b^	117.28 (10.64)^b^	14.502		<.001
Stroop Word Test (sec)	21.21 (5.38)	36.84 (7.93)^b^	42.20 (5.31) ^b^	93.497		<.001
Stroop Interference Test (sec)	32.41 (8.41)	37.32 (6.85)	49.04 (12.67)^b^	23.506		<.001

Abbreviations: AVLT, Auditory Verbal Learning Test; HAMA, Hamilton Anxiety Rating Scale; HAMD, Hamilton Depression Rating Scale; MoCA, Montreal Cognitive Assessment Test; *SD*, standard deviation; WAIS, Wechsler Adult Intelligence Scale.

^a^ Compared with HC group (*p* < .05).

^b^ Compared with HC group (*p* < .01).

^C^ Compared with VPFC group (*p* < .01).

### 2.2 Neuropsychological background tests

To assess the cognitive and emotional problems of brain tumor patients and healthy controls, we used a series of neuropsychological tests. First, the Beijing Version of the Montreal Cognitive Assessment Test (Nasreddine, 2006) was used to assess cognitive function. Second, neuropsychological functions, including attention, memory, executive function, verbal fluency, and information processing speed, were measured using the Digit Span of the Wechsler Adult Intelligence Scale (WAIS) (Wechsler, 1981), the Chinese version of the Auditory Verbal Learning Test (ALVT) (Schmidt, 1996), Stroop Color Word Test (Stroop, 1935), and the Trail Making Test (Klove, 1963). Finally, we employed HAMD and HAMA to investigate the participants' potential depression and anxiety symptoms. All assessments were administered by skilled psychologists and psychiatrists preoperatively.

### 2.3 Decision‐making tasks

#### 2.3.1 Decision‐making under ambiguity (Iowa Gambling Task)

We used the Chinese computerized version of the IGT (A. Bechara et al., 2000) to imitate decision‐making under ambiguity in real life. This task included four decks: A, B, C, and D. All participants were asked to select one card each time. After each selection, a specified amount of fictitious money, which was gained or lost, was shown on the screen. The participants were instructed to win as much as possible in 100 trials over the starting capital (¥2,000). No other information was provided on the screen besides the gain or loss after each selection and the change in money. Decks A and B were supposed to be disadvantageous options, with higher immediate gains but higher losses in the long run, and decks C and D were supposed to be the advantageous options, with little immediate returns but higher gains in the long run. We calculated the total net score by subtracting the frequency of disadvantageous selections from the frequency of advantageous selections to analyze the task performance. The net score of each block, with the 100 trials equally divided into five blocks, was calculated to investigate whether decision‐making changed over time.

#### 2.3.2 Decision‐making under risk (Game of Dice Task)

We used the computerized GDT (Brand et al., 2005) to imitate decision‐making under risk in real life. This task included eighteen trials in total and had an initial capital of ¥1,000. Before the experiment started, the rules and amounts of gains and losses were explicitly shown on the screen, and participants were instructed to win as much money as possible. For each dice, participants chose the option to have a single digit (e.g., 1, 2, 3, 4, 5, 6), two numbers (e.g., 1 and 2, 3 and 4, 5 and 6), three numbers (e.g., 1 2 3 or 4 5 6), or four numbers (e.g., 1, 2, 3, 4 or 2, 3, 4, 5 or 3, 4, 5, 6), with the probability of winning money of 1/6, 2/6, 3/6, and 4/6, respectively. Each option was associated with different winning probabilities and gains/losses. Based on the winning probability, the former two options were viewed as risky decisions and the latter two were viewed as safe decisions. We calculated how often the four different options were chosen (single, double, triple, or quadruple numbers). Moreover, the gain or loss, the changed capital, and the remaining number of dice throws were presented on the screen after each throw. We examined the use of negative feedback after selecting a disadvantageous option (one number or two numbers) in motivating the selection of an advantageous option (three or four numbers) in the subsequent trial. We defined the percentage use of positive feedback as the number of times the participant switched to an advantageous option after receiving positive feedback divided by the number of times the participant received positive feedback.

### 2.4 Statistical analysis

Statistical analysis was performed using SPSS 16. 0 (SPSS). We used ANOVA to analyze the neuropsychological test results. We then used an analysis of variance (ANOVA) test and repeated‐measures ANOVA to assess the performance differences in the decision‐making tasks. Groups were compared using Bonferroni's correction and Tamhane's tests. The threshold of statistical significance was set at *p* < .05.

## 3 RESULT

### 3.1 Neuropsychological background tests

The participants’ demographic characteristics and results from the neuropsychological tests are shown in Table 1. A one‐way ANOVA confirmed that no significant differences in age, education, the Hamilton Anxiety Rating Scale (HAMA), or the Hamilton Depression Rating Scale (HAMD) were observed between the healthy control (HC) group and the two brain tumor patient groups (VPFC and DLPFC). The two patient groups performed significantly worse on the Montreal Cognitive Assessment (MoCA), the test of attention (Stroop Color Test), the memory assessment (recognition), and the assessment of information processing (the Trail Making Test B and the Stroop Word Test) compared with the HC group. Additionally, the DLPFC group performed significantly worse on the test of assessing memory (immediate Recall, delayed Recall, and recognition tests) as well as assessing information processing (the Stroop tests) compared with the VPFC and HC groups.

### 3.2 Lesion analysis and lesion volume

**Figure 1:**
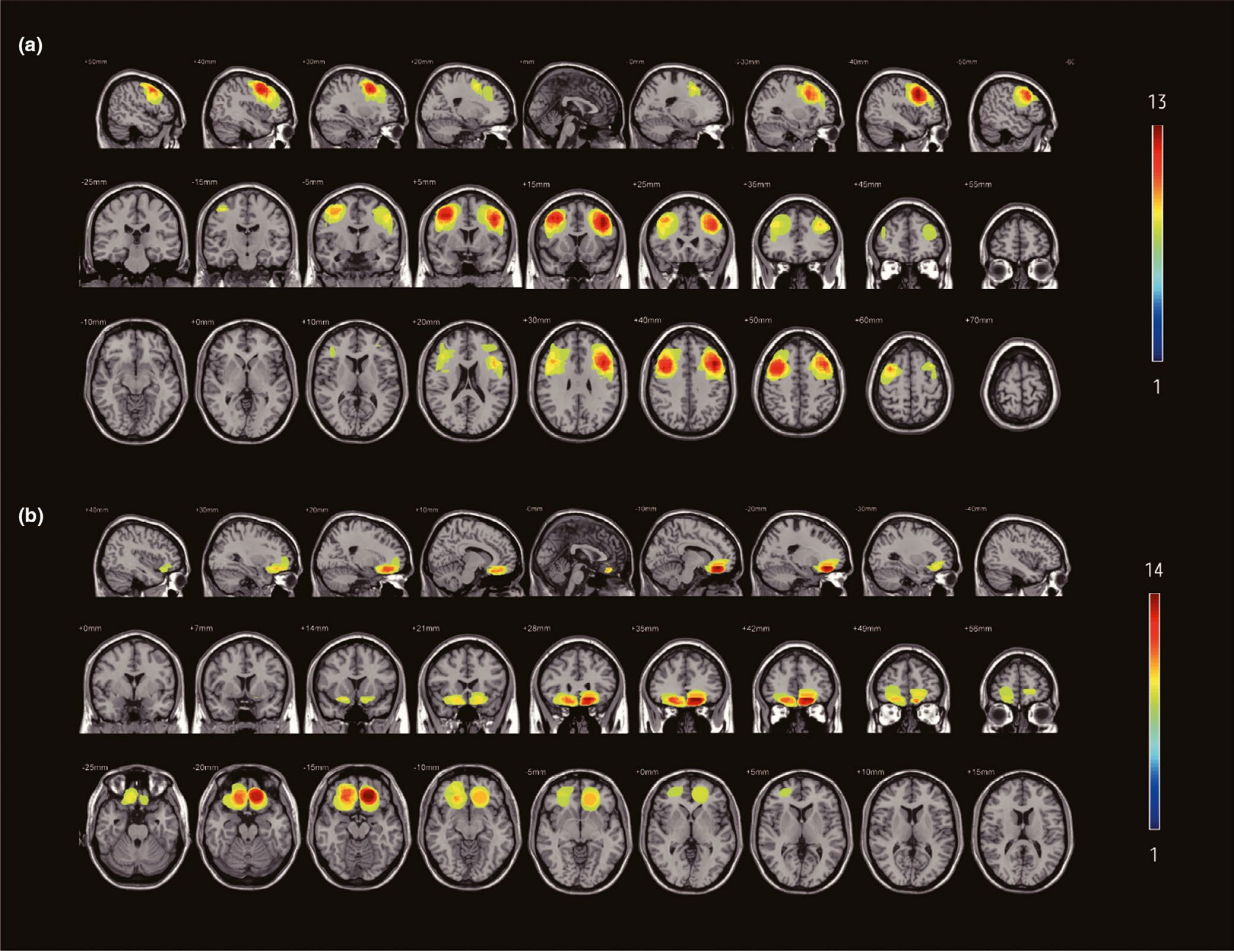
Location and degree of overlap of brain tumor. The top three rows (a) showed the location of 29 patients with DLPFC damage. The latter three rows (b) showed the location of 27 patients with VPFC damage. Lesions were projected on especially same nine axial, coronal, and sagittal slices of the standard brain. The color bars, which range from cool to warm colors, showed how many subjects had damage in the same location

According to previous MRI scans, combining the T2‐weighted scans (which generally show precise pathological information) and T1‐weighted scans (with their excellent anatomical definition), lesions were drawn using the MRIcro software (www.mricro.com) (Rorden & Brett, 2000). Anatomical lesions underlying decision‐making deficits were studied using tumor volumes, center coordinates, overlapping lesion images, and a lesion subtraction analysis provided by MRIcro software (Figure 1). Frontal‐lesion volume was significantly larger in the DLPFC group [*M* = 13,740.07 mm^3^, *SD* = 3,450.26] than in the VPFC group [*M* = 7,022.00 mm^3^, *SD* = 2,196.32] (*p* < .05). Because variables that have significant differences in the background test may interfere with performance of IGT and GDT, we conducted partial correlations with MoCA, memory function, information processing, executive function, and emotional state (HAMD and HAMA) as the control factors. There was no significant association between the frontal‐lesion volume and the performance of IGT and GDT tasks in the VPFC group (NetscoreIGT, *R* = −0.205, *p* = .415 > .05; NetscoreGDT, R = 0.085, *p* = .736 > .05), as well as in the DLPFC group (NetscoreIGT, *R* = −0.289, *p* = .217 > .05; NetscoreGDT, *R* = −0.200, *p* = .389 > .05).

### 3.3 Decision‐making on the IGT

A one‐way analysis of variance (ANOVA) was performed to examine the IGT net score and the final outcomes. The VPFC group had a significantly lower IGT net score compared with the HC group, but no significant difference was observed in the outcomes (Table 2). A repeated‐measures ANOVA was performed, with block as the within‐subjects factor and group as the between‐subjects factor. There were significant main effects for groups (*F*(2, 85) = 3.479, *p* = .035) and blocks (*F*(4, 340) = 10.978, *p* < .001). By post hoc tests (multiple comparisons) of the repeated‐measures ANOVA, different patterns of performance over the task between the VPFC group and the healthy controls (*p* = .030). There was no difference between the DLPFC group and the healthy controls (*p* = .874 > .05) as well as between the VPFC group and the DLPFC group (*p* = .373 > .05). According to pairwise comparisons on each group, the net score of the healthy controls in blocks 2 through 5 was significantly higher than the score in blocks 1 (all *p* < .05). The net score of the DLPFC group in blocks 4 and 5 were significantly higher than those in blocks 1 and 2 (all *p* < .05). However, the decision‐making process had no significant effect on the net score of the VPFC group (all *p* > .10). Pairwise comparisons of performances on the five blocks between the VPFC group and the other groups indicated significant net score differences in blocks 4 (HC group: mean = 3.813, *SD* = 1.245; VPFC group: mean = −0.370, *SD* = 1.355; DLPFC group: mean = 3.931, *SD* = 1.308; *F*(2, 87) = 3.393, *p* = .038) and block 5 (HC group: mean = 4.250, *SD* = 1.177; VPFC group: mean = −2.296, *SD* = 1.282; DLPFC group: mean = 4.138, *SD* = 1.237; *F*(2, 87) = 8.895, *p* < .001).

**Table 2:** Decision‐making performance of patients and healthy controls

	HC group (*n* = 32)	VPFC group (*n* = 27)	DLPFC group (*n* = 29)	ANOVA	
	Mean (*SD*)	Mean (*SD*)	Mean (*SD*)	*F*(2,85)	*p*
Performance					
IGT					
Total net score	11.44(19.28)	−2.00(17.59)^a^	2.93(22.80)	3.428	.037
Outcome	1624.22(316.26)	1,350.93(579.43)	1,640.52(767.05)	2.226	.114
GDT					
Total net score	9.31(5.42)	1.85(12.81)^a^	−2.14(10.45)^b^	10.696	<.001
Outcome	3.12(2088.60)	−985.19(4,459.71)	−2648.28(3,539.33)^b^	4.590	.013
Use of negative feedback (%)	62.35(33.88)	42.68(36.23)	35.97(26.38)^b^	5.230	.007
Use of positive feedback (%)	63.29(30.20)	56.19(34.43)	38.83(37.46)^a^	4.016	.022

^a^ Compared with HC group (*p* < .05).

^b^ Compared with HC group (*p* < .01).

**Figure 2:**
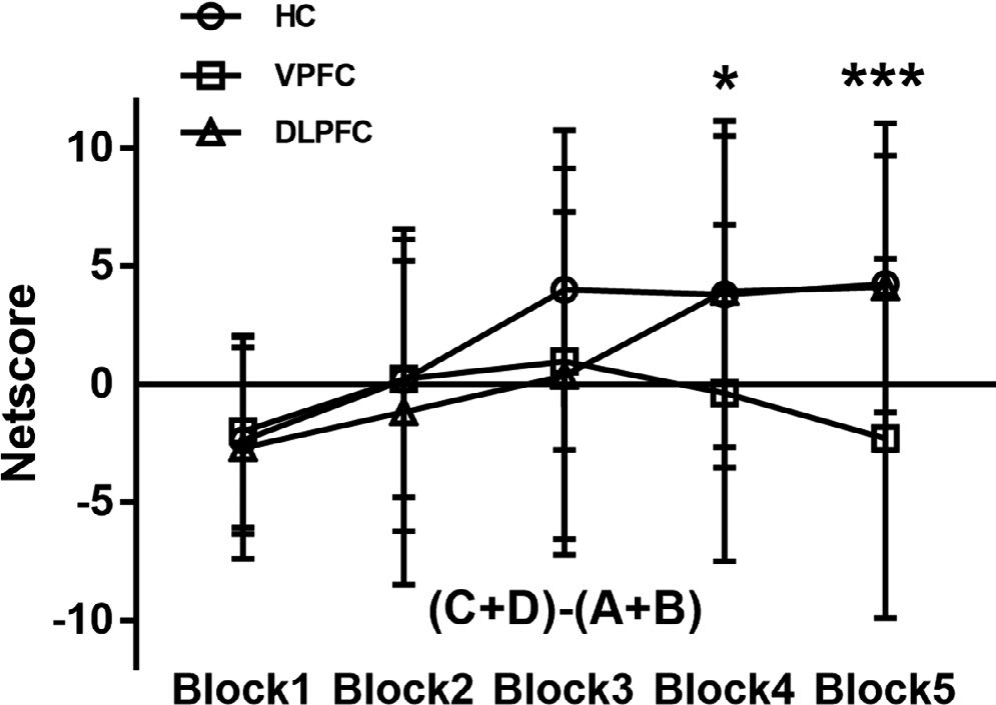
Mean net score (the number of advantageous choices (C + D) minus the number of dis‐advantageous choices (A + B)) of the five blocks in the Iowa Gambling Task (IGT) for the healthy controls, DLPFC tumor group patients, and VPFC tumor group patients [bars indicate the standard error of the mean. *p < 0. 05 for the differences between healthy controls, DLPFC tumor group, and VPFC tumor group. ***p < 0. 01 for the differences between three groups]

In general, the change curve of the net score indicated a change in the decision strategy during the IGT. As shown in Figure 2, the net score of the DLPFC group and the healthy controls markedly increased over the task, and the net score became positive in the third or fourth block, indicating that they had already turned to advantageous choices. However, the increase in the net score of the VPFC group was not obvious, indicating that they maintained the preference for disadvantageous choices.

### 3.4 Decision‐making on the GDT

**Figure 3:**
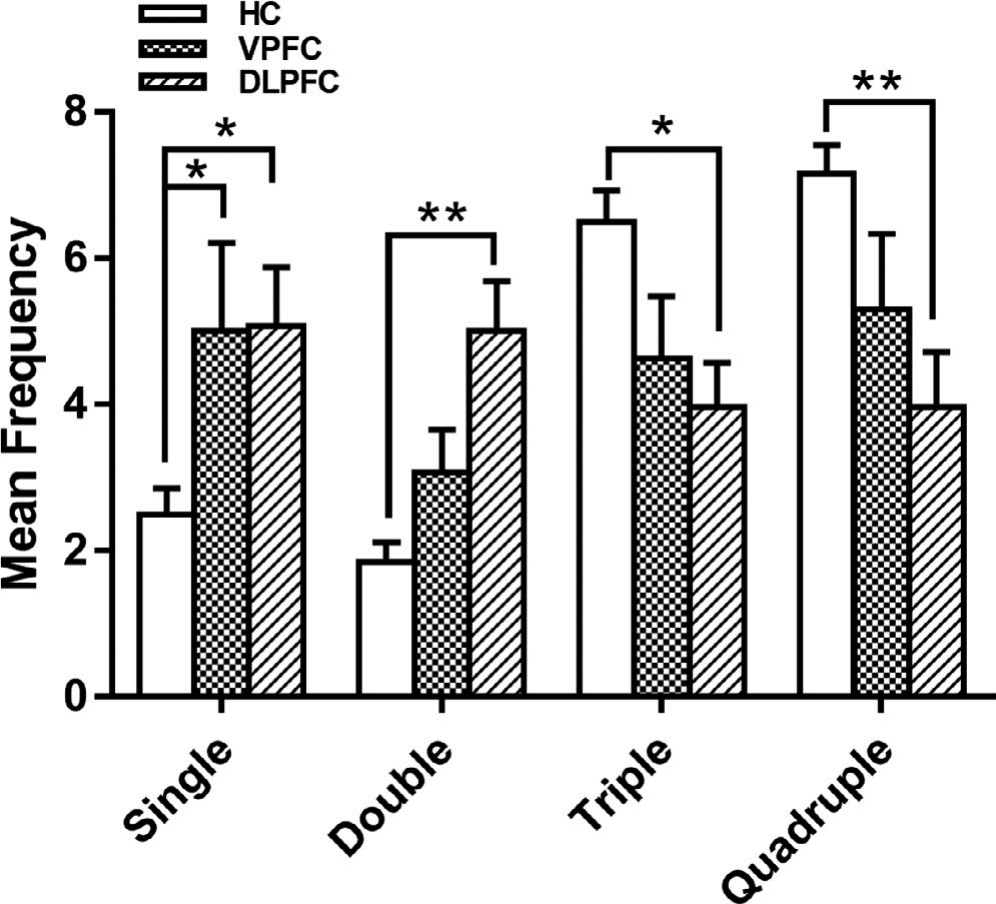
Mean frequency of each alternative in the Game of Dice Task (GDT) for the healthy controls, VPFC tumor group patients, and DLPFC tumor group patients. Significant differences were found in the frequency of choosing one number, two numbers, three numbers, and four numbers between the DLPFC tumor group and other groups [bars indicate the standard error of the mean. *means *p* < .05; **means *p* < .01]

A one‐way ANOVA was executed to examine the GDT net scores and the outcomes. There were significant differences between the net scores of the GDT among the three groups and the outcomes (Table 2). An analysis of variance with repeated measures was carried out, using choice as the within‐subjects factor and group as the between‐subjects factor. As shown in Figure 3, there was a significant main effect for choice (*F*(3, 255) = 4.475, *p* = .004) and a significant interaction between choice and group (*F*(6, 255) = 4.872, *p* < .001). Patients in the DLPFC group selected single‐ and double‐number combinations most frequently, and the healthy controls and VPFC group were more likely to select triple‐ and quadruple‐number combinations. Comparisons between the groups revealed significant differences in the frequency for choosing one single number (HC group: mean = 2.50, *SD* = 2.03; VPFC group: mean = 5.00, *SD* = 6.31; DLPFC group: mean = 5.07, *SD* = 4.38; *F*(2, 87) = 3.279, *p* = .042), double numbers (HC group: mean = 1.84, *SD* = 1.53; VPFC group: mean = 3.07, *SD* = 3.04; DLPFC group: mean = 5.00, *SD* = 3.72; *F*(2, 87) = 9.283, *p* < .001), triple numbers (HC group: mean = 6.50, *SD* = 2.44; VPFC group: mean = 4.63, *SD* = 4.43; DLPFC group: mean = 3.97, *SD* = 3.30; *F*(2, 87) = 4.509, *p* = .014), and quadruple numbers (HC group: mean = 7.16, *SD* = 2.23; VPFC group: mean = 5.30, *SD* = 5.41; DLPFC group: mean = 3.97, *SD* = 4.08; *F*(2, 87) = 4.835, *p* = .010) by (Figure 3).

**Figure 4:**
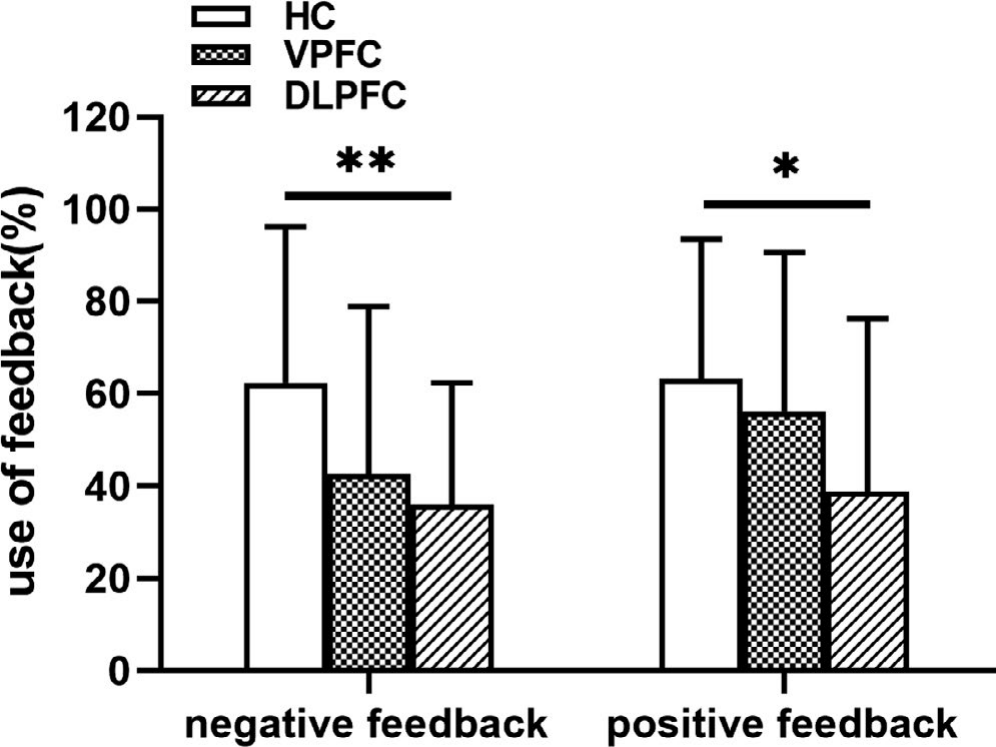
Positive and negative feedback in the Game of Dice Task for the healthy controls, VPFC tumor group patients, and DLPFC tumor group patients. Compared with the HC group, the LPFC groups made less use of negative feedback (*p* < .01) and less use of positive feedback (*p* < .05) [bars indicate the standard error of the mean. *means *p* < .05; **means *p* < .01]

We examined the use of negative feedback after selecting a disadvantageous option (one number or two numbers) in motivating the selection of an advantageous option (the combination of three or four numbers) in the subsequent trial. We defined the percentage use of positive feedback as the number of times the participant switched to an advantageous option after receiving positive feedback divided by the number of times the participant received positive feedback (Brand et al., 2005; Brand et al., 2008; Brand et al., 2008; Euteneuer et al., 2009). Here, we used a one‐way ANOVA to analyze negative/positive feedback. There was a significant group effect for negative feedback (*F*(2, 85) = 5.230, *p* = .007) and positive feedback (*F*(2, 85) = 4.016, *p* = .022). Then, multiple comparisons were made between the three groups by using Bonferroni's correction. Compared with the HC group, DLPFC groups made less use of negative feedback and positive feedback (*p* < .05), meaning that the patients continued to make disadvantageous choices. Conversely, healthy controls more often changed their behavior to select an advantageous option for their next choice (Figure 4).

It has been reported that the decision‐making function is affected by different cerebral hemispheres. Compared with the right DLPFC, the left DLPFC participates in IGT function processing and significantly improves the decision‐making function after anode electrical stimulation (He et al., 2016). In this study, we compared the scores of neuropsychological background tests and performance of IGT and GDT in the left and right DLPFC by independent sample *t* test, and found no significant difference between the two groups (all *p* > .05).

## 4 DISCUSSION

To our knowledge, few studies have directly investigated the two types of decision‐making functions in patients with brain tumors in different brain regions. This study mainly found that the decision‐making functions of subjects in the VPFC and DLPFC tumor groups were impaired to varying degrees. Among them, there was decision‐making impairment under ambiguity and under risk in the VPFC group, as well as decision‐making impairment under risk in the DLPFC group. In addition, the results of neuropsychological tests showed that the two tumor groups performed significantly worse on attention, memory, information processing, and executive function compared with the HC group. Furthermore, patients in the DLPFC group performed significantly worse on memory and information processing assessments compared with the VPFC and HC groups.

We found that performance on the IGT and GDT in the VPFC tumor group was significantly impaired compared with that of the HC group. As the change curve of the net score indicated a change in decision strategy during the IGT, after the initial exploration and understanding of the rules, the DLPFC tumor group and the control group began to choose favorable options with stable benefits and continued using this strategy, while those in the VPFC tumor group could not correctly evaluate the short‐term and long‐term benefits and continued to make disadvantageous choices. Most previous studies have acknowledged that the VPFC (with the limbic system as the main structure, including the ventral and medial sectors of the prefrontal cortex, insular cortex, striatum, amygdala, and parietal cortex) plays a crucial role in the IGT (Bechara & Martin, 2004

) and accordingly propose the somatic labeling hypothesis. Bechara et al. held the view that VPFC injury patients could not generate larger anticipatory SCRs when they selected from a risky deck compared with when they selected from a safe deck (Bechara et al.,^1996^). A number of neuroimaging studies have also proven activation of the medial frontal cortex during various decisions under uncertainty (Ernst et al., 2002; Fukui et al., 2005; Li et al., 2010). It was found that the VPFC group had different degrees of damage. Although there are not enough data to support the VPFC participating in decision‐making under risk, we believe the deviation may be caused by the following reasons. First, potential brain overlap, such as the ACC/striatum, may be an important factor (Botvinick, 2007). Second, like the impairment of attention, memory, information, and emotional processing shown in our research, alterations of other cognitive functions would affect decision‐making under ambiguity. According to the abovementioned properties, we believe that damage of the VPFC with the limbic system as the core mainly impairs the decision‐making function under ambiguity, but the impairment of other cognitive functions due to the involvement of overlapping brain regions ultimately affects the decision‐making function under risk.However, we found that the DLPFC group was only impaired in decision‐making in a gambling situation with explicit and stable rules for gains and losses, whereas healthy participants showed risk avoidance. “The dorsolateral prefrontal loop” (Crutcher & Alexander, 1990), which mainly comprises the dorsolateral prefrontal cortex and lateral orbitofrontal cortex with the caudate nucleus, has been demonstrated to be involved in GDT processing and plays a major role in decision‐making under risk. Some research indicates that deciding advantageously in a decision‐making task with explicit and stable rules was linked to applying calculative strategies as a part of executive function (Brand et al., 2008; Brand, Roth‐Bauer, et al., 2008). In contrast, individuals who decide intuitively prefer risky or disadvantageous choices on the GDT (Brand et al., 2008a; Brand, Roth‐Bauer, et al., 2008). Ultimately, numerous neuroimaging studies have shown that the DLPFC is crucial for executive function (Charlton et al., 2008; Ko et al., 2008; Niendam et al., 2012), and perfect executive function is the guarantee for the realization of other cognitive processes (Gouveia et al., 2007). Furthermore, the DLPFC group had lower positive and negative feedback utilization rates, resulting in an impaired risky decision‐making function. It is possible that damage of the pallium in the DLPFC or a decrease in connection density with other brain regions led to an interruption in the loop connection (A. Bechara et al., 2003). However, some studies have also suggested the involvement of more extensive structures in the IGT, including the dorsolateral prefrontal cortex (Brand et al., 2007; Manes et al., 2002). Research now considers that the IGT is a process from an ambiguity decision to a risk decision (Brand et al., 2008b); the continuous and dynamic process of the IGT requires the collaborative activities of the VPFC and DLPFC (Fellows & Farah, 2005b; Lawrence et al., 2009). However, the cohort data analysis of this study did not support that the decision‐making function of the DLPFC group was impaired under ambiguity and only a small percentage of patients had impairment that may have been caused by the population heterogeneity of brain tumor patients and the experimental design. Therefore, in future studies, we hope to further explore how the DLPFC regulates the decision‐making function by increasing the sample size and using different techniques (such as fMRI and other neuroimaging technologies).

There were some limitations to our current study. First, this study mainly focused on the influence of prefrontal tumors on decision‐making function and did not include patients with other brain tumors as the control group. Although we tried to exclude patients with HAMD > 7 and/or HAMA > 7, research still needs to rule out emotional changes caused by tumors, which led to dysfunctions of cognitive behavior and decision‐making function. Future research attempted to include other brain lesions as control group, to explore the impact of prefrontal tumors on decision‐making function. Moreover, because of this study's small sample size, the brain tumor pathology was not used as a variable to explain whether tumors of different properties at the same site caused analogous changes in the decision‐making function. Second, our goal was to recruit patients with prefrontal tumors with relatively focal damage compared with other research studies. Due to brain edema, irregular shape of tumors, and other reasons, our data of overlapping showed some outliers in each group. Third, this study was based entirely on behavior, and the mechanism can be explored by combining neuroimaging and electrophysiological technologies in the future. Finally, these experimental defects prompted us to further increase the sample size and explore the potential neural mechanism of the decision‐making network by combining functional neuroimaging, behavioral, and disease model studies.

## 5 CONCLUSION

In summary, VPFC and DLPFC damage could lead to impairment in the decision‐making function and other cognitive functions. Based on prefrontal brain tumor patients, these results indicate that the internal neural mechanism of decision‐making is complicated and advantageous decision‐making behavior requires the joint participation of the VPFC and DLPFC. Further research is needed to verify how the VPFC and DLPFC regulate decision‐making behavior under risk and ambiguity. This study has the potential to provide insight into the brain processes underlying decision‐making behaviors in a variety of pathological conditions.

## CONFLICT OF INTEREST

The authors declare no conflict of interest.

## AUTHOR CONTRIBUTIONS

Kai Wang and Xingui Chen designed the study and supervised the data collection. Yuyang Wang initiated the study and drafted the manuscript. Xukou Wang was involved in data collection. Xingui Chen and Bing Zhao critically revised the manuscript.

### Peer Review

The peer review history for this article is available at https://publons.com/publon/10.1002/brb3.1951.

## Data Availability

The data of this study are available upon reasonable request.
